# A six-year epidemiological study of selected zoonotic abortifacient agents in ovine and caprine foetuses in Türkiye

**DOI:** 10.1017/S0950268824001699

**Published:** 2024-12-19

**Authors:** Murat Şevik

**Affiliations:** Department of Virology, Veterinary Faculty, Necmettin Erbakan University, Konya, Türkiye

**Keywords:** abortion, Cache valley virus, *Chlamydia abortus*, *Coxiella burnetii*, *Listeria monocytogenes*

## Abstract

Abortion is one of the major threats to the livestock industry, and it also poses significant threats to public health since some of the abortifacient agents are considered zoonotic. *Chlamydia abortus* (*C. abortus*), *Coxiella burnetii* (*C. burnetii*), *Listeria monocytogenes* (*L. monocytogenes*), and Cache Valley virus (CVV) are recognized as important zoonotic and abortifacient agents of reproductive failure in small ruminants. This study determined the prevalence of these agents in ovine and caprine foetuses in Türkiye. A total of 1 226 foetuses were collected from the sheep (n = 1 144) and goats (n = 82) from different flocks between 2012 and 2017. Molecular detection methods were used to detect *C. abortus*, *C. burnetii*, and *L. monocytogenes* DNA and CVV RNA in aborted foetuses. In this study, *C. abortus* was the most prevalent abortifacient agent among the investigated ovine (264/1144) and caprine (12/82) foetuses, followed by *C. burnetii* with a frequency of 2.8% (32/1144) and 8.5% (7/82) in ovine and caprine foetuses, respectively. *L. monocytogenes* DNA was detected in 28 (2.4%) and 2 (2.4%) of the ovine and caprine foetuses, respectively. However, CVV RNA was not detected. Although the predominant mixed infection was *C. abortus* and *C. burnetii*, mixed infection of *C. abortus* and *L. monocytogenes*, and *C. burnetii* and *L. monocytogenes* were also found. The information presented in this study contributes to the understanding of the roles of *C. abortus*, *C. burnetii*, *L. monocytogenes*, and CVV in abortions in small ruminants, and could be beneficial for developing more effective control strategies.

## Introduction

Abortion is one of the major problems for the livestock industry that affects the reproductive and productive performance of small ruminants. The causes of abortion can be categorized into non-infectious causes (genetic and physical factors, nutritional and metabolic problems, heat stress, and toxic agents) and infectious agents [1]. The major infectious agents associated with abortion are bacteria (such as *Brucella* spp., *Campylobacter* spp., *Chlamydia abortus* (*C. abortus*), *Coxiella burnetii* (*C. burnetii*), and *Listeria monocytogenes* (*L. monocytogenes*)), viruses (such as pestiviruses, bluetongue virus, Schmallenberg virus, and Cache valley virus (CVV)), and parasites (such as *Neospora caninum* and *Toxoplasma gondii*) [1, 2]. Although non-infectious causes are related to lower abortion rates, infectious agents cause severe abortion outbreaks [1]. Infectious agents are also significant threats to public health since most of the abortifacient agents are considered zoonotic [1, 2]. Zoonotic potencies of abortifacient agents such as *C. abortus*, *C. burnetii*, *L. monocytogenes*, and CVV have been described [3–6].

*Chlamydia abortus* and *C. burnetii* are obligate intracellular zoonotic Gram-negative bacteria and have been associated with reproductive problems in livestock and pregnant women [1, 3, 6]. Previous studies in pregnant women have found that *C. abortus* infection occurs in two cases per year in the UK [6], whereas *C. burnetii* infection occurs in at least one in every 540 pregnancies in Southern France [3]. The prevalence of *C. abortus* and *C. burnetii* among sheep and goat flocks ranged from 2.0% to 43.3% [7–11] and 2.0% to 49.0% [11–14], respectively. Ramo et al. [15] reported that *C. abortus* and *C. burnetii* were identified in approximately 75% of ovine and caprine abortions in Spain. Furthermore, previous studies have also found that *C. abortus* and *C. burnetii* are the main abortive agents in small ruminants in Algeria [16], Ethiopia [17], Hungary [18], and Iran [19]. In Türkiye, the proportion of abortive episodes potentially related to *C. abortus* was 7.7% in sheep and 14.3% in goats [8, 20], whereas the detection rate of *C. burnetii* was 6.1% in sheep and 8.5% in goats foetal samples analyzed between 2019 and 2020 [13].

*Listeria monocytogenes* is a motile, facultative intracellular Gram-positive bacterium. Various clinical manifestations can be seen in infected animals such as encephalitis, keratitis, uveitis, septicaemia, late gestation abortion, and stillbirth, whereas it can cause septicaemia, meningitis, miscarriage, and stillbirth in humans [5]. *L. monocytogenes*-related foetal losses have been reported in pregnant women in Denmark, Iran, and the USA [21, 22, 23]. The epidemiological studies revealed that *L. monocytogenes* have been reported as a cause of abortion among small ruminants in Austria (25%) [24], Iraq (20.3%) [25], Denmark (8.3%) [26], and India (2.8%) [27]. Although the prevalence of *L. monocytogenes* in caprine foetuses in Türkiye is unknown, limited numbers of studies have investigated the role of *L. monocytogenes* in ovine foetuses in Türkiye, and it has been reported that the detection rate of *L. monocytogenes* in ovine foetuses ranged from 2.8% to 13.3% [28, 29].

Cache valley virus is a neurotropic arbovirus that can cause congenital defects, stillbirth, and spontaneous abortion in small ruminants [1, 30]. Furthermore, CVV infection has been associated with encephalitis and meningitis in humans [4]. CVV infection has not yet been reported in Türkiye. However, serological and molecular evidence of CVV infection in small ruminants has been reported in a few countries such as the USA [30], Canada [31], and Mexico [32].

Determining the cause of abortion in ruminants is difficult due to the complex aetiology of abortion. Previous studies conducted in Türkiye have reported the presence of zoonotic abortifacient agents in goats and sheep [20, 33, 34]. Despite the previous studies that demonstrated the presence of zoonotic abortifacient agents among small ruminants, the prevalence of the zoonotic abortifacient agents is largely unknown at the national level in Türkiye because previous studies were mostly conducted in a small area or a limited number of flocks. Furthermore, many studies on major infectious agents causing abortions in small ruminants in Türkiye rely on serological tests only [33, 35, 36]. This can be misleading to determine the actual cause of abortion. Identifying the aetiologic agent of abortion has important implications for developing effective flock health strategies, and also for the prevention and control of zoonotic diseases [16, 17]. Therefore, the current study aimed to investigate the prevalence of important zoonotic abortifacient agents in small ruminants in Türkiye.

## Methods

### Study area

**Figure 1.**
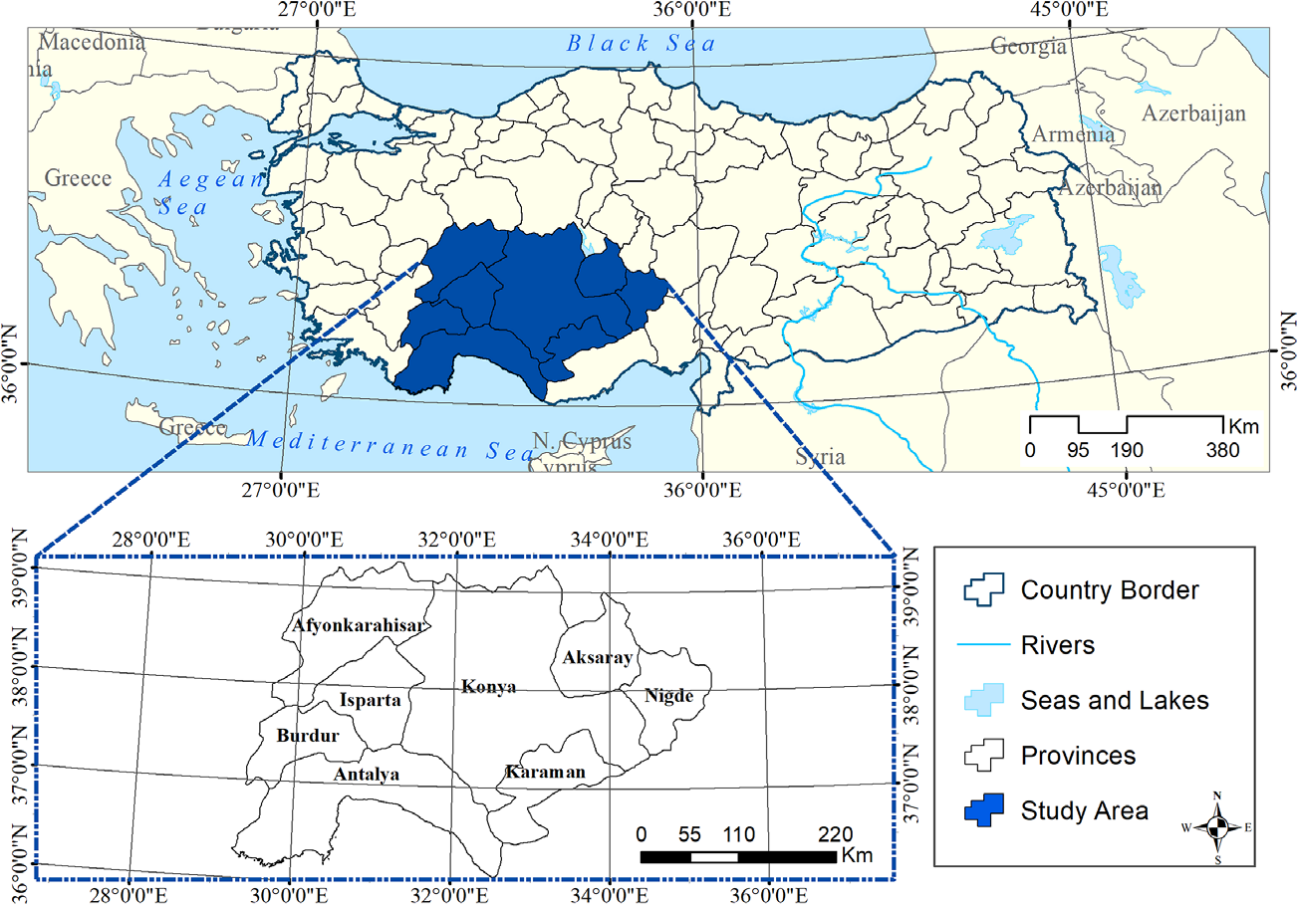
Map of Türkiye showing the sampled provinces.

Türkiye has seven geographical regions. The present study was performed in three different geographical regions of Türkiye where livestock production is one of the main sources of income, including the Mediterranean region (Isparta, Burdur, and Antalya Provinces), the Aegean region (Afyonkarahisar Province), and the Central Anatolian region (Niğde, Aksaray, Karaman, and Konya Provinces) (Figure 1). These regions had 5 012 677 sheep and 1 714 788 goats. The small ruminant industry in surveyed regions is dominated by small-scale family flocks (n = 10–50) and settled village flocks (n = 100–500). White Karaman and Dağliç breeds of sheep and Hair goat breed of goats were common in surveyed regions.

### Clinical samples

A total of 1 226 foetuses (1 144 sheep and 82 goats) were submitted to the Veterinary Control Institute (Konya, Türkiye) during 2012–2017 from different flocks (n = 1 226) with abortion histories. These investigated flocks constitute about 0.005% of all the sheep and goat flocks in surveyed regions. Aborted foetuses were submitted within 24 h after abortion. Unfortunately, the placenta samples were not submitted to the institute in most of the abortion cases. Therefore, only aborted foetuses were used to determine the cause of abortion.

To avoid cross-contamination, a necropsy was done using sterile surgical instruments under biosafety guidelines. During the necropsy, foetal tissues were obtained from foetuses with gestational age < 3 months, while organ samples, including lymph nodes, liver, lung, kidney, intestine, and spleen were obtained from foetuses with gestational age 3 to 5 months. Each animal’s samples were put in sterile 50 ml falcon tubes and were kept at −85 °C until analysis.

Furthermore, a questionnaire was administered to farmers to obtain information related to abortion cases, the clinical signs, the number of pregnant animals on the flock, the number of animals that had been aborted, and the date of abortion.

### Nucleic acid extraction

Pooled tissue/organ samples of each foetus (30–60 mg) were placed into sterile DNAse/RNAse-free microcentrifuge tubes (2 ml) containing phosphate-buffered saline (400 μl) and homogenized using the TissueRuptor (Qiagen, Germany). Total nucleic acids were extracted from the supernatants of the centrifuged foetal tissue homogenates (5 000 g for 5 min at 4 °C) using the QIAamp Cador Pathogen Mini Kit (Qiagen, Germany), according to protocols reported by the kit manufacturers, and were stored at −85 °C until analysis.

### Molecular identification of abortifacient agents

Samples were tested for the presence of *C. abortus*, *C. burnetii*, *L. monocytogenes*, and CVV by molecular detection methods. For *C. abortus*, *C. burnetii*, and *L. monocytogenes*, real-time PCR assays reported by Pantchev et al. [37], Klee et al. [38], and Rossmanith et al. [39] were used, respectively. Furthermore, one-step real-time duplex RT-PCR was performed using CVV G1 glycoprotein-specific probes and primers described by Wang et al. [40]. Negative control (sterile nuclease-free water) was used to verify the absence of cross-contamination during the analyzes, whereas positive controls were used to ensure that results were reliable.

### Statistical analysis

The differences in positivity between species (sheep and goats) were assessed by Fisher’s exact test, whereas one-way ANOVA with Tukey post-test was used to assess the relationship between positivity and provinces and years of origin. The statistical analyzes were performed by using the GraphPad Prism software (San Diego, CA, USA), and a p-value of ≤  0.05 was considered statistically significant.

## Results

### Questionnaire survey results

According to flock owners’ reports, sampled flocks had not been vaccinated against investigated diseases, and animals exhibited no distinct symptoms, except for abortion. Abortions mostly occurred during the last trimester of gestation. The rate of abortion in *C. abortus*, *C. burnetii*, and *L. monocytogenes* positive flocks ranged from 8.6% to 46.5%, 7.2% to 10.5%, and 6.3% to 9.7%, respectively.

### Detection of abortifacient agents

*Chlamydia abortus* DNA was detected in 276 (22.5%) of the 1 226 aborted foetuses with 23.1% (264/1144) positive ovine foetuses and 14.6% (12/82) positive caprine foetuses. There was no significant difference in the positivity of *C. abortus* between ovine and caprine foetuses (p = 0.099). The highest number of *C. abortus*-positive foetuses was detected in 2016 (41.3%) (p = 0.006), followed by 2015 (28.3%) (Table 1). The number of *C. abortus*-positive foetuses (88/277, 31.8%) was significantly higher in Konya Province than in Isparta and Antalya Provinces (p = 0.03). Aksaray Province had the second highest rate, with 30.9% (29/94), followed by 27.4% (20/73) in Afyonkarahisar, 23.1% (9/39) in Karaman, 20.8% (59/284) in Niğde, 19.2% (10/52) in Burdur, 15.3% (23/150) in Isparta, and 14.8% (38/257) in Antalya Province.

**Table 1.** The prevalence of *C. abortus*, *C. burnetii*, *L. monocytogenes*, and CVV in ovine and caprine foetuses in the study area during 2012–2017

	Ovine foetuses																		Caprine foetuses																	
	2012			2013			2014			2015			2016			2017			2012			2013			2014			2015			2016			2017		
Pathogen	N	P	%	N	P	%	N	P	%	N	P	%	N	P	%	N	P	%	N	P	%	N	P	%	N	P	%	N	P	%	N	P	%	N	P	%
*C. abortus*	176	11	6	155	29	19	294	54	18	343	103	30	118	53	45	58	14	24	4	1	25	5	–	–	5	–	–	31	3	10	25	6	24	12	2	17
*C. burnetii*	176	8	5	155	–	–	294	10	3	343	7	2	118	7	6	58	–	–	4	–	–	5	–	–	5	–	–	31	1	3	25	3	12	12	3	25
*L. monocytogenes*	176	7	4	155	–	–	294	7	2	343	13	4	118	1	1	58	–	–	4	1	25	5	–	–	5	–	–	31	–	–	25	1	4	12	–	–
CVV	176	–	–	155	–	–	294	–	–	343	–	–	118	–	–	58	–	–	4	–	–	5	–	–	5	–	–	31	–	–	25	–	–	12	–	–

*Source:* CVV = Cache valley virus, N = number of tested samples, P = number of positive samples, % = prevalence rate, and any fractional part greater than or equal to 0.5 was rounded up to the next whole number.

*Coxiella burnetii* positivity was significantly higher in caprine foetuses (8.5%, 7/82) than in ovine foetuses (2.8%, 32/1144) (p = 0.012). The number of *C. burnetii*-positive foetuses (5.1%, 2/39) was higher in Karaman Province than in other provinces, without significant differences. Konya Province had the second highest rate, with 5.0% (14/277), followed by 3.5% (10/284) in Niğde, 3.2% (3/94) in Aksaray, 2.7% (2/73) in Afyonkarahisar, 2.0% (3/150) in Isparta, and 1.9% (5/257) in Antalya Province. However, *C. burnetii* was not detected in samples from Burdur Province (0/52).

*Listeria monocytogenes* DNA was detected in 28 (2.4%) and 2 (2.4%) of the 1 144 ovine, and 82 caprine foetuses, respectively. There was no significant difference in the positivity of *L. monocytogenes* between ovine and caprine foetuses (p = 1.000). The highest *L. monocytogenes*-positive foetuses were detected in Aksaray Province (5/94, 5.3%), without significant differences, followed by 3.6% (10/277) in Konya, 2.7% (2/73) in Afyonkarahisar, 2.7% (7/257) in Antalya, 2.6% (1/39) in Karaman, and 1.8% (5/284) in Niğde Province. However, *L. monocytogenes* was not detected in samples from Burdur Province (0/52) and Isparta Province (0/152).

CVV RNA was not detected in ovine and caprine foetuses.

In this study, although the predominant mixed infection was *C. abortus* and *C. burnetii* (sheep, n = 4; goat, n = 1), mixed infection of *C. abortus* and *L. monocytogenes* (sheep, n = 1), and *C. burnetii* and *L. monocytogenes* (sheep, n = 1) were also found.

## Discussion

Abortions in small ruminants have important impacts on livestock production, and non-infectious and infectious agents can cause abortion in ruminants [2]. Many abortifacient agents in small ruminants pose a serious health threat to humans [1]. The exact prevalence of common zoonotic and abortifacient agents in Türkiye is still unclear because the diagnostic testing of abortifacient agents in small ruminants in Türkiye relies mostly on serological tests. Therefore, in this study prevalence of major zoonotic and abortifacient agents was investigated. To the best of my knowledge, this study is the longest and most comprehensive study that investigated the prevalence of zoonotic agents in ovine and caprine foetuses.

WOAH recommended the molecular diagnostic assays to detect the aetiologic agents of disease [41]. Therefore, molecular diagnostic assays were used for the detection of *C. abortus*, *C. burnetii*, *L. monocytogenes*, and CVV in aborted foetuses.

In this study, abortion-related pathogens were detected in 28.1% (345/1226) of the cases. However, an agent could not be detected in 71.9% of the cases, which may either be related to other infectious agents that were not examined in this study or non-infectious factors such as genetic factors, toxins, stress, nutritional, and hormonal problems [14].

In this study, the detection rate of *C. abortus* in ovine foetuses (23.1%) was higher than that reported in previous studies from Türkiye (range from 3.5% to 7.7%) [8, 10, 20], Iran (12.3%) [14], and Italy (2%) [7], but was lower than detection rates from Spain (38.3%) [11] and Algeria (43.3%) [9]. Possible explanations for this discrepancy may be the number of sampled flocks and animals, the difference in sampling technique, the diagnostic technique used, and the lack of strategies to prevent and control *C. abortus* infection. There is no control programme for *C. abortus* infection at the national level in Türkiye. Furthermore, the higher detection rate of *C. abortus* in ovine foetuses observed in this study can be explained by the flock management practices. The introduction of animals with unknown health status is considered a significant risk factor for *C. abortus* infection [19]. However, in the study area, it was common to introduce purchased animals from flocks with unknown health status to the flock without any testing or quarantine. This could be one of the factors associated with the higher detection rate of *C. abortus* in this study. Another reason for the higher detection rate of *C. abortus* in this study could be the mineral nutrition deficiencies. Tejedor-Junco MT et al. [42] reported that proper nutrition is a protective factor against *C. abortus* infection. In most flocks in the studied regions, the system of sheep management is extensive. The extensive management system is more likely to lead to mineral nutrition deficiencies. Mineral nutrition deficiencies can cause an impaired immune response and loss of host resistance to infection [43].

In this study, the detection rate of *C. abortus* in caprine foetuses (14.6%) is in agreement with a previous report from Eastern Türkiye that approximated detection rate of 14.3% [8], but was lower than that detected (21.4%) in a previous study from the Marmara region of Türkiye [10]. This result can be explained by the fact that goat farming in the Marmara region is generally under intensive and semi-intensive production systems, which facilitates the spread of *C. abortus* infection. It has been reported that the disease spreads faster when animals are overcrowded, as the contact between healthy and infected animals increases in intensive and semi-intensive flocks [9].

This study shows no significant differences in the positivity of *C. abortus* between ovine and caprine foetuses (p = 0.099), indicating that both sheep and goats are equally susceptible to the disease. This finding is in agreement with the results of a previous study from Spain [11].

In this study, the highest *C. abortus* rate was recorded in Konya Province (31.8%). In Konya Province, the main sheep breed is Merino, which lambs year-round. Lambing throughout the year can cause the pathogen to circulate continuously within the flock, which can be a constant source of infection [44]. Furthermore, the average flock size (greater than 152 sheep) in Konya Province was larger than other sheep populations in other studied provinces. A significant positive association between *C. abortus* seropositivity and larger flock size has been reported [45]. Sheep overcrowding in large size flocks can have an impact on animal welfare and hygiene, which increases the risk of *C. abortus* transmission [17, 42].

In this study, the detection rate of *C. abortus* increased from 2012 to 2016, and it tended to decline in 2017 (Table 1). Possible explanations for this discrepancy may be the increasing knowledge of *C. abortus* infection and awareness of good hygienic measures among farmers.

In this study, the detection rate of *C. burnetii* in ovine foetuses (2.8%) is in agreement with previous studies from Türkiye that reported *C. burnetii* detection rate in ovine foetuses ranges between 2.0% and 2.9% [12, 46], but the detection rate in this study was lower than that observed in previous studies that reported detection rate ranges from 6.1% to 49.0% in ovine foetuses [11, 13, 14, 34]. Possible explanations for this discrepancy may be the difference in husbandry practices, and the sample type. According to the farmers’ report, pregnant animals were separated from the rest of the flock during parturition, placentas, aborted foetuses, and uterine fluids were removed by burning. The transmission of the disease in the flock can be controlled by implementing these measures [18]. Furthermore, in this study, only, foetal tissue samples were used to detect *C. burnetii* DNA. However, it has been reported that placental tissue is more useful for the detection of *C. burnetii* nucleic acids [11].

In this study, the detection rate of *C. burnetii* in caprine foetuses (8.5%) is in agreement with a previous report from Türkiye [13], but was lower than the 40.0% reported by Günaydin et al. [34]. This variation could be related to the number of sampled animals (82 in this study vs. 5 in Günaydin et al. [34]), the sample type (foetal tissues and organ samples in this study vs. abomasal contents in Günaydin et al. [34]), the diagnostic technique used (real-time PCR in this study vs. conventional PCR in Günaydin et al. [34]), the hygiene conditions, and the presence of tick species which are capable of transmitting *C. burnetii.* Ticks are believed to be the vectors for *C. burnetii* transmission to animals, and *C. burnetii* is most commonly present in *Ixodes*, *Racicephalus*, *Dermacentor*, and *Haemaphysalis* genera [46, 47]. The tick species, *Hyalomma marginatum*, *Hyalomma anoliticum excavatum*, *Hyalomma detritum*, and *Boophilus annulatus* belonging to the *Haemaphysalis* and *Ixodes* genera, that can transmit *C. burnetii* to animals have been detected in the Black Sea region of Türkiye [46], where a higher detection rate (40.0%) of *C. burnetii* was reported by Günaydin et al. [34].

In this study, the highest *C. burnetii* detection rate (7/82, 8.5%) was observed in caprine foetuses. This finding is in agreement with previous studies that reported *C. burnetii*-related abortions are more common in goats than in sheep [13, 34]. However, detection rates of *C. burnetii* in sheep and goats may not be due to differences in susceptibility to the infection. Because the precise time of the entry of *C. burnetii* onto the flock or the route of transmission was not known in the investigated flocks.

In this study, the highest *C. burnetii* rate was recorded in Karaman Province (2/39, 5.1%). This result can be explained by the flock type. Sheep and goats are often raised together in mixed flocks in Karaman Province. The higher prevalence of *C. burnetii* in mixed flocks has also been reported in Germany and Italy [44, 48]. It has been reported that keeping sheep and goats in the same flock may increase animal density and increase the chances of contact between animals, thus facilitating cross-transmission between sheep and goats [17].

Unfortunately, there is no control programme against *C. burnetii* infection in Türkiye, and this leads to the persistence of the infection (Table 1).

The detection rate of *L. monocytogenes* (2.4%) in this study is in agreement with a previous study that reported the detection rate of *L. monocytogenes* in ovine foetuses was 2.8% in Türkiye [29]. However, a lower detection rate was found in this study compared with previous studies that reported the detection rate of *L. monocytogenes* rates in ovine foetuses ranged from 9.0% to 13.3% [28, 49]. Furthermore, the detection rate of *L. monocytogenes* in caprine foetuses (2.4%) was lower than that reported in previous studies that *L. monocytogenes* rates in aborted caprine foetuses ranged from 8.0% to 16.2% [49, 50]. Feed quality and storage, farm management practices, animal health, and hygienic conditions are all possible explanations for this discrepancy. *L. monocytogenes* is commonly found in poorly fermented silage, and there is a causal relationship between the prevalence of listeriosis in ruminants and the consumption of poorly fermented silage [51]. Akca et al. [28] reported that the high-detection rate of *L. monocytogenes* in ovine foetuses in their study may be due to the consumption of low-quality silage feed. In the regions being studied, the traditional method of raising sheep and goats involves extensive farming, and the animals’ nutrition is largely dependent on meadows and pastures. Nightingale KK et al. [51] reported that access to pasture is a protective factor against *L. monocytogenes* infection. Furthermore, in this study sampled animals exhibited no distinct symptoms, except for abortion. However, Kim et al. [50] collected aborted foetuses from the flocks that had neurological symptoms associated with listeriosis. This might be the explanation for the higher detection rate of *L. monocytogenes* in caprine foetuses in their study.

In this study, the highest *L. monocytogenes* rate was recorded in Aksaray Province (5.3%, 5/94), whereas *L. monocytogenes* was not detected in samples from Burdur and Isparta Provinces. This variation could be related to the number of sampled animals and flocks, differences in animal population density in sampled provinces, and the strains of *L. monocytogenes.* The virulence potential of *L. monocytogenes* strains varies. While some of the *L. monocytogenes* strains are non-virulent and unable to cause infection, others are virulent and cause high mortality and morbidity rates [50]. In this study, the virulence characterization of *L. monocytogenes* strains circulating in the study area was not performed. However, the circulation of virulent *L. monocytogenes* strains in Aksaray Province has been reported [52]. This could explain the highest detection rate of *L. monocytogenes* in ovine and caprine foetuses in the Aksaray Province.

The detection rate of *L. monocytogenes* increased from 2013 to 2015, and it tended to decline in 2016 (Table 1). This observation can be explained by increased awareness and improvement of hygiene measures on flocks in response to the worsening situation with peste des petits ruminants (PPR) in the study area. PPR outbreaks were observed in the study area in 2015 (WAHIS database), and control measures, including quarantine and hygiene measures, were applied within the study area. Hygienic measures were found to be a significant tool in reducing the spread of *L. monocytogenes* on farms [51].

In this study, the predominant mixed infections were *C. abortus* and *C. burnetii.* This finding is similar to previous studies where *C. burnetii* and *C. abortus* coinfection were the most frequently diagnosed in ovine and caprine foetuses [11, 18]. This result suggests that pathogen synergism could play a role as an abortigenic agent in some abortions associated with *C. burnetii* infection.

There is no report of CVV-associated cases in Türkiye. However, new arboviral diseases are emerging in new geographical regions as a result of climate change [31]. Therefore, the presence of CVV was investigated in this study. In this study, CVV RNA was not also detected in aborted foetuses. CVV is transmitted by mosquitoes and biting midges, and it is widely distributed in North America [30, 31, 40]. Therefore, not detection of CVV in this study may be related to the climatic conditions and geographical distribution of vector populations involved in the transmission of CVV [40].

This study has some limitations. First, data was gathered through self-administered questionnaires, which could lead to bias in reporting. Second, being conducted in three different geographical regions of Türkiye, there are concerns regarding geographical variation, and the representativeness of obtained data for all regions of Türkiye is uncertain. Third, other infectious agents causing abortions in sheep and goats, such as *Brucella* spp., *Campylobacter* spp., *Toxoplasma gondii*, and *Neospora caninum*, were not investigated in this study due to budgetary limits. This limitation should be taken into account when interpreting the findings of the study. Finally, genotyping of the detected isolates was not performed. A future study should assess the genotypes of these isolates to provide more comprehensive data on *C. abortus*, *C. burnetii*, and *L. monocytogenes* epidemiology.

## Conclusions

*Chlamydia abortus* was the most frequently detected abortifacient agent in the 1 226 abortion cases in the evaluation period, with a frequency of 22.5%. To a lesser extent, *C. burnetii* (3.3%), and *L. monocytogenes* (2.4%) were also detected in these abortion cases. The findings of the present study suggest that *C. abortus*, *C. burnetii*, and *L. monocytogenes* should be taken into consideration in abortion cases of small ruminants in the surveyed provinces. To implement effective control strategies against diseases that cause abortion in small ruminants, it is necessary to determine the infectious agents circulating in the field. Therefore, the results of this study should be taken into account in the development of control and protection strategies for abortion in small ruminants. Furthermore, these diseases have zoonotic potential, and therefore, farmers should take hygienic precautionary measures when handling aborted materials.

## Data Availability

The data presented in this study are available within the article.
